# 2-Cyano-*N*′-(2-hy­droxy-3-meth­oxy­benzyl­idene)acetohydrazide

**DOI:** 10.1107/S1600536811025451

**Published:** 2011-07-09

**Authors:** Hongbo Li, Peng Chen

**Affiliations:** a College of Chemistry and Biology Engineering, Yancheng Institute of Technology, Yancheng 224051, People’s Republic of China

## Abstract

The title compound, C_11_H_11_N_3_O_3_, was obtained by the reaction of 3-meth­oxy­salicyl­aldehyde with cyano­acetohydrazide in methanol. There is an intra­molecular O—H⋯N hydrogen bond in the mol­ecule. In the crystal, mol­ecules are linked by N—H⋯O hydrogen bonds, generating chains running along the *b* axis.

## Related literature

For the structures of hydrazones, see: Wang *et al.* (2011 ▶); Hashemian *et al.* (2011 ▶); Singh & Singh (2010 ▶); Ahmad *et al.* (2010 ▶).
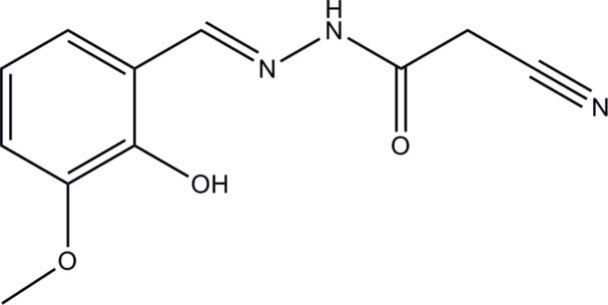


## Experimental

### 

#### Crystal data

C_11_H_11_N_3_O_3_
*M* *_r_* = 233.23
Orthorhombic, 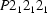

*a* = 4.8035 (14) Å
*b* = 9.470 (3) Å
*c* = 23.884 (7) Å
*V* = 1086.5 (5) Å^3^
*Z* = 4
Mo *K*α radiation
μ = 0.11 mm^−1^
*T* = 298 K
0.23 × 0.18 × 0.17 mm

#### Data collection

Bruker SMART 1K CCD area-detector diffractometer
Absorption correction: multi-scan (*SADABS*; Sheldrick, 2004 ▶) *T*_min_ = 0.976, *T*_max_ = 0.982
6959 measured reflections
2298 independent reflections
1606 reflections with *I* > 2σ(*I*)
*R*_int_ = 0.058

#### Refinement

*R*[*F* ^2^ > 2σ(*F* ^2^)] = 0.061
*wR*(*F* ^2^) = 0.114
*S* = 1.04
2298 reflections
159 parameters
1 restraint
H atoms treated by a mixture of independent and constrained refinement
Δρ_max_ = 0.18 e Å^−3^
Δρ_min_ = −0.22 e Å^−3^

### 

Data collection: *SMART* (Bruker, 2001 ▶); cell refinement: *SAINT* (Bruker, 2001 ▶); data reduction: *SAINT*; program(s) used to solve structure: *SHELXTL* (Sheldrick, 2008 ▶); program(s) used to refine structure: *SHELXTL*; molecular graphics: *SHELXTL*; software used to prepare material for publication: *SHELXTL*.

## Supplementary Material

Crystal structure: contains datablock(s) I, global. DOI: 10.1107/S1600536811025451/qm2013sup1.cif

Structure factors: contains datablock(s) I. DOI: 10.1107/S1600536811025451/qm2013Isup2.hkl

Supplementary material file. DOI: 10.1107/S1600536811025451/qm2013Isup3.cml

Additional supplementary materials:  crystallographic information; 3D view; checkCIF report

## Figures and Tables

**Table 1:** Hydrogen-bond geometry (Å, °)

*D*—H⋯*A*	*D*—H	H⋯*A*	*D*⋯*A*	*D*—H⋯*A*
N2—H2*A*⋯O3^i^	0.90 (1)	2.20 (2)	2.995 (3)	148 (3)
O2—H2⋯N1	0.82	1.91	2.626 (3)	145

Symmetry code: (i) 
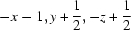
.

**Fig. 1.**
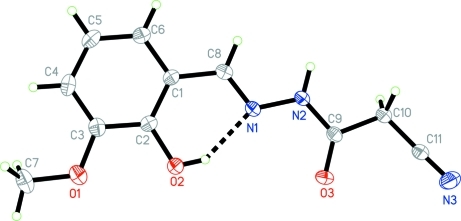
The molecular structure of (I), with atom labels and 30% probability displacement ellipsoids for non-H atoms. Intramolecular O—H···N hydrogen bond is shown as a dashed line.

**Fig. 2.**
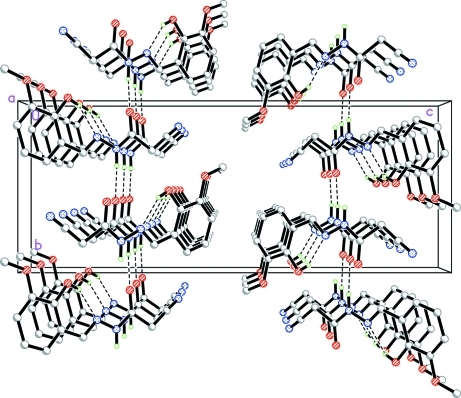
The packing of (I), viewed down the a axis. Hydrogen bonds are shown as dashed lines. H atoms not involved in hydrogen bonding have been omitted.

## supplementary crystallographic information

### Comment

Recently, a great number of hydrazones derived from the reaction of salicylaldehyde and its derivatives with benzohydrazides (Wang *et al.*, 2011; Hashemian *et al.*, 2011; Singh & Singh, 2010; Ahmad *et al.*, 2010). To the best of our knowledge, the hydrazones derived from cyanoacetohydrazide have never been reported so far. In this paper, the title new hydrazone compound, (I), is reported.

There is an intramolecular O—H···N hydrogen bond (Table 1) in the molecule of (I), Fig. 1. The non-hydrogen atoms of the compound are approximately coplanar, with mean deviation from the least-squares plane of 0.026 (3) Å. In the crystal structure, molecules are linked by N—H···O hydrogen bonds (Table 1), generating chains running along the *b* axis (Fig. 2).

### Experimental

The title compound was obtained by the reaction of equimolar quantities (1.0 mmol each) of 3-methoxysalicylaldehyde with cyanoacetohydrazide in methanol. Single crystals suitable for X-ray diffraction were obtained by the slow evaporation of the solution containing the compound in open air.

### Refinement

H atoms were positioned geometrically and refined using a riding model, with C—H = 0.93–0.97 Å, O—H = 0.82 Å, and with *U*_iso_(H) = 1.2 (1.5 for methyl group and O) times *U*_eq_(C).

### Crystal data

**Table:**

C_11_H_11_N_3_O_3_	*D*_x_ = 1.426 Mg m^−^^3^
*M**_r_* = 233.23	Mo *K*α radiation, λ = 0.71073 Å
Orthorhombic, *P*2_1_2_1_2_1_	Cell parameters from 1239 reflections
*a* = 4.8035 (14) Å	θ = 2.4–24.5°
*b* = 9.470 (3) Å	µ = 0.11 mm^−^^1^
*c* = 23.884 (7) Å	*T* = 298 K
*V* = 1086.5 (5) Å^3^	Block, colorless
*Z* = 4	0.23 × 0.18 × 0.17 mm
*F*(000) = 488	

### Data collection

**Table:**

Bruker SMART 1K CCD area-detector diffractometer	2298 independent reflections
Radiation source: fine-focus sealed tube	1606 reflections with *I* > 2σ(*I*)
graphite	*R*_int_ = 0.058
ω scans	θ_max_ = 27.0°, θ_min_ = 2.3°
Absorption correction: multi-scan (*SADABS*; Sheldrick, 2004)	*h* = −6→6
*T*_min_ = 0.976, *T*_max_ = 0.982	*k* = −12→11
6959 measured reflections	*l* = −30→26

### Refinement

**Table:**

Refinement on *F*^2^	Primary atom site location: structure-invariant direct methods
Least-squares matrix: full	Secondary atom site location: difference Fourier map
*R*[*F*^2^ > 2σ(*F*^2^)] = 0.061	Hydrogen site location: inferred from neighbouring sites
*wR*(*F*^2^) = 0.114	H atoms treated by a mixture of independent and constrained refinement
*S* = 1.04	*w* = 1/[σ^2^(*F*_o_^2^) + (0.0452*P*)^2^] where *P* = (*F*_o_^2^ + 2*F*_c_^2^)/3
2298 reflections	(Δ/σ)_max_ < 0.001
159 parameters	Δρ_max_ = 0.18 e Å^−^^3^
1 restraint	Δρ_min_ = −0.22 e Å^−^^3^

### Special details

**Table:**

Geometry. All e.s.d.'s (except the e.s.d. in the dihedral angle between two l.s. planes) are estimated using the full covariance matrix. The cell e.s.d.'s are taken into account individually in the estimation of e.s.d.'s in distances, angles and torsion angles; correlations between e.s.d.'s in cell parameters are only used when they are defined by crystal symmetry. An approximate (isotropic) treatment of cell e.s.d.'s is used for estimating e.s.d.'s involving l.s. planes.
Refinement. Refinement of *F*^2^ against ALL reflections. The weighted *R*-factor *wR* and goodness of fit *S* are based on *F*^2^, conventional *R*-factors *R* are based on *F*, with *F* set to zero for negative *F*^2^. The threshold expression of *F*^2^ > σ(*F*^2^) is used only for calculating *R*-factors(gt) *etc*. and is not relevant to the choice of reflections for refinement. *R*-factors based on *F*^2^ are statistically about twice as large as those based on *F*, and *R*- factors based on ALL data will be even larger.

### Fractional atomic coordinates and isotropic or equivalent isotropic displacement parameters (Å^2^)

**Table:**

	*x*	*y*	*z*	*U*_iso_*/*U*_eq_	
N1	−0.1059 (5)	0.2286 (2)	0.20121 (9)	0.0327 (6)	
N2	−0.2854 (5)	0.2894 (2)	0.23995 (10)	0.0357 (6)	
N3	−0.9714 (7)	0.1500 (3)	0.37043 (13)	0.0710 (10)	
O1	0.4649 (5)	−0.0612 (2)	0.06598 (9)	0.0509 (6)	
O2	0.1150 (5)	0.02087 (19)	0.14348 (9)	0.0437 (6)	
H2	−0.0004	0.0580	0.1641	0.066*	
O3	−0.4697 (5)	0.0798 (2)	0.26659 (8)	0.0458 (6)	
C1	0.2477 (6)	0.2652 (3)	0.13270 (11)	0.0300 (7)	
C2	0.2719 (6)	0.1226 (3)	0.11849 (11)	0.0324 (7)	
C3	0.4616 (6)	0.0810 (3)	0.07771 (12)	0.0385 (8)	
C4	0.6298 (7)	0.1801 (3)	0.05190 (13)	0.0415 (8)	
H4	0.7581	0.1522	0.0249	0.050*	
C5	0.6059 (7)	0.3220 (3)	0.06656 (13)	0.0425 (8)	
H5	0.7195	0.3885	0.0493	0.051*	
C6	0.4189 (6)	0.3643 (3)	0.10575 (12)	0.0377 (7)	
H6	0.4041	0.4595	0.1148	0.045*	
C7	0.6697 (8)	−0.1109 (4)	0.02719 (13)	0.0619 (11)	
H7A	0.6369	−0.0693	−0.0089	0.093*	
H7B	0.6578	−0.2118	0.0243	0.093*	
H7C	0.8518	−0.0848	0.0402	0.093*	
C8	0.0503 (6)	0.3143 (3)	0.17435 (12)	0.0351 (7)	
H8	0.0371	0.4106	0.1817	0.042*	
C9	−0.4540 (6)	0.2074 (3)	0.27092 (11)	0.0316 (7)	
C10	−0.6117 (7)	0.2931 (3)	0.31379 (12)	0.0392 (8)	
H10A	−0.4798	0.3346	0.3398	0.047*	
H10B	−0.7074	0.3697	0.2949	0.047*	
C11	−0.8123 (7)	0.2116 (3)	0.34494 (13)	0.0412 (8)	
H2A	−0.287 (8)	0.3840 (11)	0.2408 (13)	0.080*	

### Atomic displacement parameters (Å^2^)

**Table:**

	*U*^11^	*U*^22^	*U*^33^	*U*^12^	*U*^13^	*U*^23^
N1	0.0339 (14)	0.0275 (12)	0.0369 (14)	0.0044 (12)	0.0030 (13)	−0.0002 (11)
N2	0.0430 (16)	0.0253 (12)	0.0388 (14)	0.0027 (13)	0.0097 (13)	−0.0028 (12)
N3	0.083 (3)	0.0503 (18)	0.080 (2)	−0.0227 (18)	0.035 (2)	−0.0092 (16)
O1	0.0569 (16)	0.0422 (12)	0.0537 (14)	0.0071 (10)	0.0123 (12)	−0.0065 (10)
O2	0.0443 (14)	0.0327 (11)	0.0541 (15)	0.0032 (10)	0.0151 (11)	0.0019 (9)
O3	0.0572 (15)	0.0251 (11)	0.0550 (13)	−0.0026 (10)	0.0130 (12)	−0.0022 (9)
C1	0.0288 (17)	0.0325 (16)	0.0286 (15)	−0.0016 (13)	−0.0042 (14)	0.0050 (13)
C2	0.0312 (18)	0.0338 (16)	0.0322 (16)	−0.0022 (14)	−0.0034 (14)	0.0037 (13)
C3	0.038 (2)	0.0424 (17)	0.0352 (17)	0.0060 (15)	−0.0015 (15)	0.0005 (15)
C4	0.0328 (18)	0.061 (2)	0.0308 (16)	0.0050 (16)	0.0044 (16)	0.0033 (15)
C5	0.038 (2)	0.0487 (19)	0.0411 (19)	−0.0096 (15)	0.0006 (16)	0.0101 (15)
C6	0.0377 (18)	0.0363 (15)	0.0389 (17)	−0.0017 (15)	−0.0015 (16)	0.0039 (14)
C7	0.067 (3)	0.067 (2)	0.052 (2)	0.010 (2)	0.008 (2)	−0.0204 (18)
C8	0.038 (2)	0.0296 (14)	0.0381 (17)	0.0015 (13)	−0.0017 (15)	−0.0016 (13)
C9	0.0327 (18)	0.0301 (16)	0.0319 (16)	0.0041 (13)	−0.0035 (14)	−0.0012 (13)
C10	0.0435 (19)	0.0307 (16)	0.0434 (18)	−0.0014 (15)	0.0096 (16)	−0.0058 (14)
C11	0.047 (2)	0.0310 (16)	0.0454 (19)	−0.0010 (15)	0.0064 (18)	−0.0085 (15)

### Geometric parameters (Å, °)

**Table:**

N1—C8	1.278 (3)	C3—C4	1.384 (4)
N1—N2	1.390 (3)	C4—C5	1.393 (4)
N2—C9	1.344 (4)	C4—H4	0.9300
N2—H2A	0.896 (10)	C5—C6	1.358 (4)
N3—C11	1.138 (4)	C5—H5	0.9300
O1—C3	1.376 (3)	C6—H6	0.9300
O1—C7	1.431 (4)	C7—H7A	0.9600
O2—C2	1.361 (3)	C7—H7B	0.9600
O2—H2	0.8200	C7—H7C	0.9600
O3—C9	1.215 (3)	C8—H8	0.9300
C1—C2	1.396 (4)	C9—C10	1.510 (4)
C1—C6	1.404 (4)	C10—C11	1.442 (4)
C1—C8	1.451 (4)	C10—H10A	0.9700
C2—C3	1.391 (4)	C10—H10B	0.9700
			
C8—N1—N2	115.8 (2)	C5—C6—H6	119.8
C9—N2—N1	120.1 (2)	C1—C6—H6	119.8
C9—N2—H2A	124 (2)	O1—C7—H7A	109.5
N1—N2—H2A	116 (2)	O1—C7—H7B	109.5
C3—O1—C7	117.5 (2)	H7A—C7—H7B	109.5
C2—O2—H2	109.5	O1—C7—H7C	109.5
C2—C1—C6	119.1 (3)	H7A—C7—H7C	109.5
C2—C1—C8	122.1 (2)	H7B—C7—H7C	109.5
C6—C1—C8	118.8 (3)	N1—C8—C1	121.6 (3)
O2—C2—C3	118.0 (2)	N1—C8—H8	119.2
O2—C2—C1	122.1 (2)	C1—C8—H8	119.2
C3—C2—C1	119.9 (2)	O3—C9—N2	124.4 (3)
O1—C3—C4	124.5 (3)	O3—C9—C10	124.1 (3)
O1—C3—C2	115.3 (3)	N2—C9—C10	111.4 (2)
C4—C3—C2	120.1 (3)	C11—C10—C9	113.4 (2)
C3—C4—C5	119.6 (3)	C11—C10—H10A	108.9
C3—C4—H4	120.2	C9—C10—H10A	108.9
C5—C4—H4	120.2	C11—C10—H10B	108.9
C6—C5—C4	120.8 (3)	C9—C10—H10B	108.9
C6—C5—H5	119.6	H10A—C10—H10B	107.7
C4—C5—H5	119.6	N3—C11—C10	178.2 (3)
C5—C6—C1	120.4 (3)		

### Hydrogen-bond geometry (Å, °)

**Table:**

*D*—H···*A*	*D*—H	H···*A*	*D*···*A*	*D*—H···*A*
N2—H2A···O3^i^	0.90 (1)	2.20 (2)	2.995 (3)	148 (3)
O2—H2···N1	0.82	1.91	2.626 (3)	145

Symmetry codes: (i) −*x*−1, *y*+1/2, −*z*+1/2.
